# Quinone binding site in a type VI sulfide:quinone oxidoreductase

**DOI:** 10.1007/s00253-022-12202-8

**Published:** 2022-10-11

**Authors:** Nikolett Miklovics, Ágnes Duzs, Fanni Balogh, Gábor Paragi, Gábor Rákhely, András Tóth

**Affiliations:** 1 Institute of Biophysics, Biological Research Centre, Szeged, Hungary; 2 Department of Biotechnology, University of Szeged, Szeged, Hungary; 3 Doctoral School in Biology, University of Szeged, Szeged, Hungary; 4 Institute of Physics, University of Pécs, Pécs, Hungary; 5 Department of Theoretical Physics, University of Szeged, Szeged, Hungary

**Keywords:** Sulfide:quinone oxidoreductase (SQR), Disulfide reductase, Sulfur metabolism, Quinone binding site, Quinone reduction

## Abstract

**Abstract:**

Monotopic membrane-bound flavoproteins, sulfide:quinone oxidoreductases (SQRs), have a variety of physiological functions, including sulfide detoxification. SQR enzymes are classified into six groups. SQRs use the flavin adenine dinucleotide (FAD) cofactor to transfer electrons from sulfide to quinone. A type VI SQR of the photosynthetic purple sulfur bacterium, *Thiocapsa roseopersicina* (TrSqrF), has been previously characterized, and the mechanism of sulfide oxidation has been proposed. This paper reports the characterization of quinone binding site (QBS) of TrSqrF composed of conserved aromatic and apolar amino acids. Val331, Ile333, and Phe366 were identified near the benzoquinone ring of enzyme-bound decylubiquinone (dUQ) using the TrSqrF homology model. In silico analysis revealed that Val331 and Ile333 alternately connected with the quinone head group via hydrogen bonds, and Phe366 and Trp369 bound the quinones via hydrophobic interactions. TrSqrF variants containing alanine (V331A, I333A, F366A) and aromatic amino acid (V331F, I333F, F366Y), as well as a C-terminal α-helix deletion (CTD) mutant were generated. These amino acids are critical for quinone binding and, thus, catalysis. Spectroscopic analyses proved that all mutants contained FAD. I333F replacement resulted in the lack of the charge transfer complex. In summary, the interactions described above maintain the quinone molecule’s head in an optimal position for direct electron transfer from FAD. Surprisingly, the CTD mutant retained a relatively high level of specific activity while remaining membrane-anchored. This is a unique study because it focuses on the QBS and the oxidative stage of a type VI sulfide-dependent quinone reduction.

**Key points:**

*• V331, I333, F366, and W369 were shown to interact with decylubiquinone in T. roseopersicina SqrF*

*• These amino acids are involved in proper positioning of quinones next to FAD*

*• I333 is essential in formation of a charge transfer complex from FAD to quinone*

**Graphical abstract:**

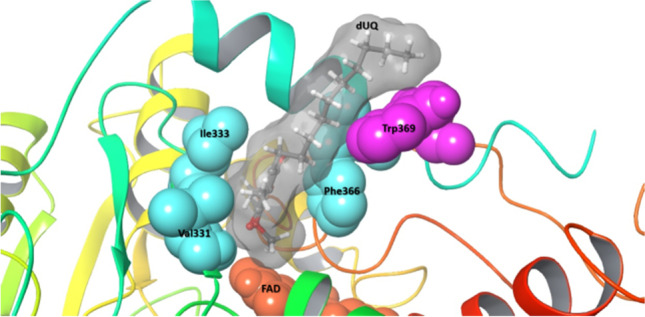

**Supplementary Information:**

The online version contains supplementary material available at 10.1007/s00253-022-12202-8.

## Introduction

Sulfide:quinone oxidoreductase (SQR) enzymes are membrane-bound flavoproteins that catalyze the oxidation of sulfide. Sulfide is the most reduced and reactive form of sulfur, making it an extremely toxic compound for cells. However, sulfide can be used as an electron and energy source by chemotrophic and photosynthetic microorganisms that tolerate higher S^2−^concentrations (Griesbeck et al. 2000; Sousa et al. 2018). In eukaryotes, it may act as a signaling molecule (Kolluru et al. 2013; Shen et al. 2016). SQRs are critical enzymes in controlling cellular sulfide levels. As a result, these enzymes participate in various physiological processes, including sulfide detoxification, microbial energy conservation, and neuronal, vascular, and smooth muscle functions in mammals (Hosoki et al. 1997; Yan et al. 2004).

**Fig. 1:**
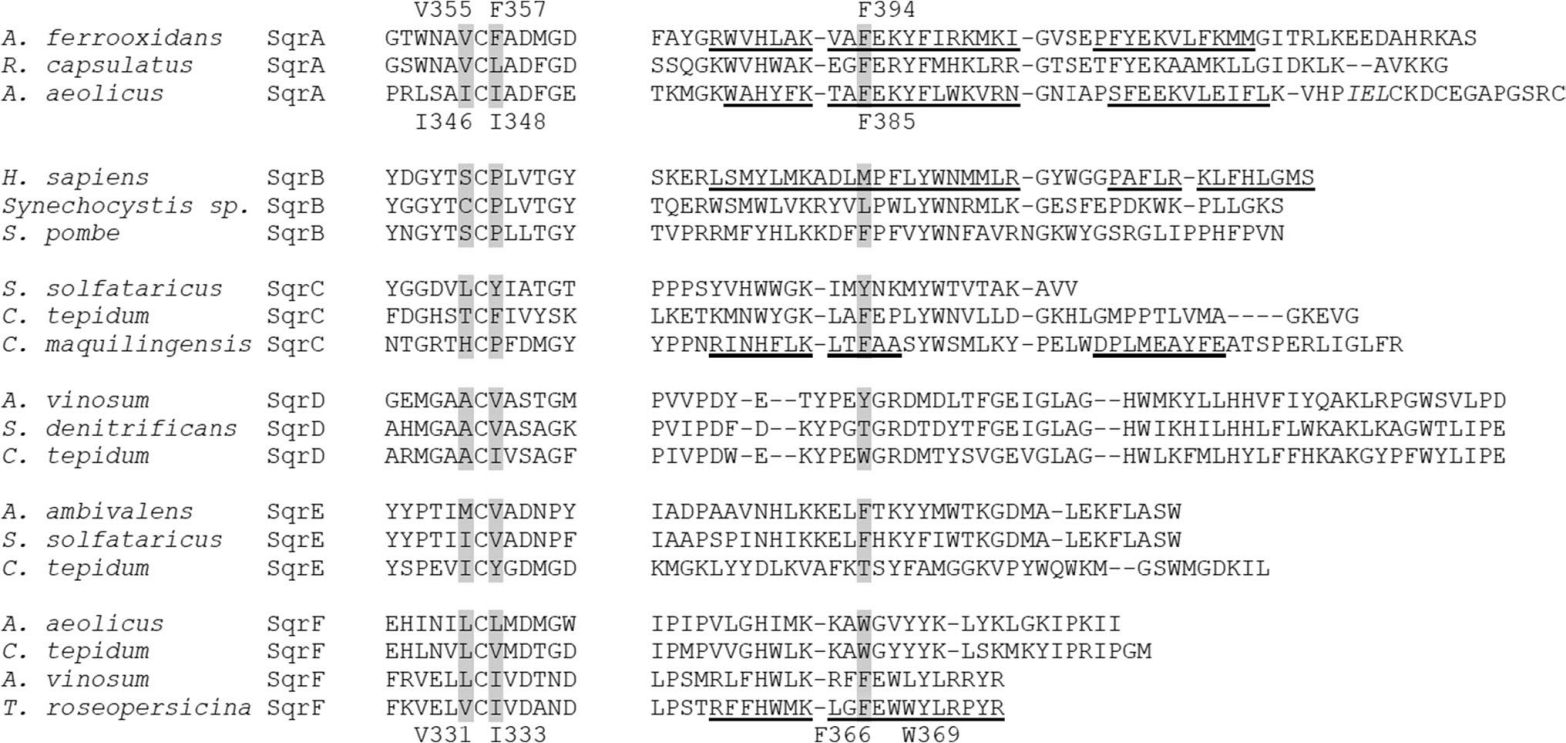
Multiple amino acid sequence alignment of two segments of the C-terminal domains of several representative SQR enzymes harboring amino acids involved in the formation of the quinone channel (highlighted in the gray background) (*A. ferrooxidans*, *A. aeolicus*, and *T. roseopersicina* residue numbering). The amino acids located in the α-helices of SQRs’ C-terminal domain are underlined

The SQR proteins may have evolved during the development of the first living organisms that required these enzyme activities in the sulfidic anoxic environment of ancient oceans (Theissen et al. 2003; Marcia et al. 2010a). These enzymes are now found in many bacteria and archaea but are particularly prevalent in eukaryotes (Sousa et al. 2018). Based on phylogenetic sequence analysis and structural alignments, SQR enzymes have been classified into six groups (type I–VI, SqrA–F) (Marcia et al. 2010a; Shuman and Hanson 2016; Sousa et al. 2018). SQRs are members of a functionally diverse group of redox enzymes known as the “two dinucleotide binding domains flavoprotein” (tDBDF) superfamily, which also includes protein families such as ferredoxin reductases, glutathione reductases, dihydrolipoamide dehydrogenases, type II NADH:quinone oxidoreductases, and sulfide:quinone oxidoreductase (Sousa et al. 2017). All tDBDF proteins, including SQRs, contain three domains: two structural scaffold Rossmann fold domains evolved for the specific binding of dinucleotide cofactors (flavin adenine dinucleotide [FAD] and NAD[P]H) and a smaller C-terminal domain (Ojha et al. 2007). The first Rossmann fold in SQRs contains the FAD cofactor, bound via non-covalent interactions. The other Rossmann structure is primarily responsible for the catalytic site for sulfide binding and oxidation. In some enzymes, it also binds the cofactor via forming a covalent bond with the isoalloxazine ring of FAD (Duzs et al. 2021). It has been reported that the C-terminal domain, which is composed of β-sheets and α-helical structural elements, plays a role in membrane binding and protein oligomerization (Lencina et al. 2013, 2020). Additionally, this domain is responsible for hosting the binding site for the enzyme’s electron acceptor quinone substrates (Marcia et al. 2009, 2010a; Cherney et al. 2010; Zhang et al. 2016). The C-terminal domain of most SQRs contains two amphipathic α-helices except for members of type VI group, which have a shorter C-terminal domain consisting of only one terminal α-helix (Duzs et al. 2021) (Fig. 1).

SQR catalyzes a complex two-step redox reaction in which electrons are transferred from sulfide to the respiratory and photosynthetic membrane electron flow in microorganisms and mitochondria via their FAD cofactor (Brito et al. 2009; Bauzá et al. 2013). The first reductive phase of the redox reaction is the two-electron oxidation of sulfide to elemental sulfur combined with the reduction of FAD, resulting in the formation of an octameric sulfur ring (S^8^) or a linear polysulfide chain (HS-(S_n_)-SH) after several catalytic cycles by coupling the oxidized sulfur atoms. The second oxidative half-reaction involves the transfer of two electrons from the reduced FAD cofactor to a lipophilic electron carrier quinone molecule located within the cytoplasmic membrane of prokaryotes and the inner mitochondrial membrane of eukaryotes (Cherney et al. 2012).

**Fig. 2:**
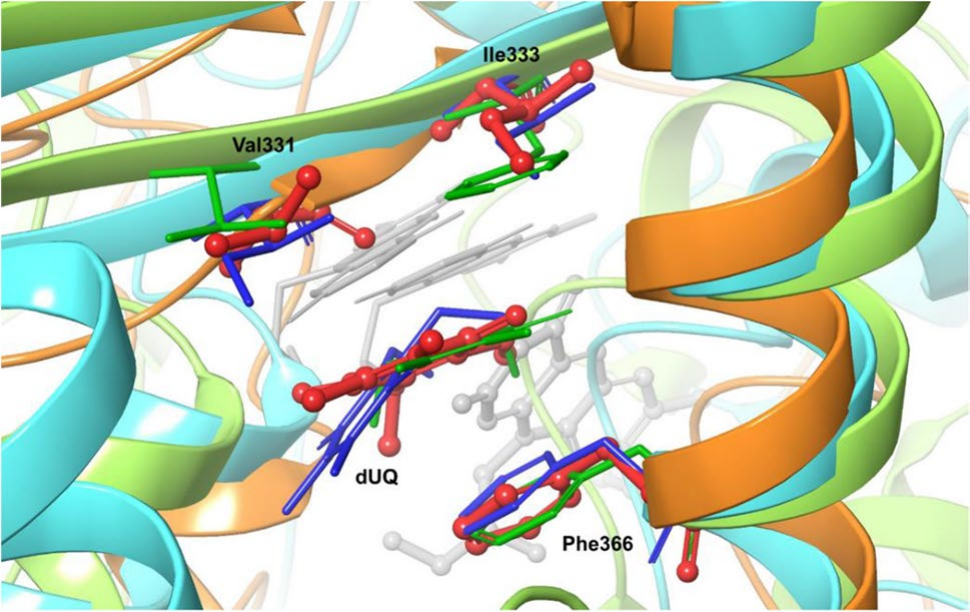
Structural alignment of the quinone-binding sites of *A. ferrooxidans*, *A. aeolicus*, and *T. roseopersicina* SQR enzymes. Structures were aligned by fitting the *α* and *β* carbons of *T. roseopersicina* Val331, Ile333, and Phe366 and their corresponding residues in SqrA of *A. ferrooxidans* (Val, Phe, Phe) and *A. aeolicus* (Leu, Leu, Trp) using the Maestro GUI program from the Schrödinger suit (Schrödinger Release 2022–1). The protein backbones are depicted in a new cartoon ribbon representation (light green: X-ray structure of *A. ferrooxidans* SqrA (PDB ID: 3T31); light blue: X-ray structure of *A. aeolicus* SqrA (PDB ID: 3HYW); orange: homology model of TrSqrF (Duzs et al. 2021)). The apolar amino acids that form the QBS and the bound dUQ in *A. ferrooxidans* (colored green) and *A. aeolicus* SqrA (colored blue) are represented by sticks. The amino acids in the corresponding positions and the dUQ ligand in the SqrF protein of *T. roseopersicina* are denoted by stick and ball marks (colored in red) and are numbered. The decyl side chain of dUQ molecules is absent. Additionally, the FAD cofactor is depicted as light gray sticks (in *A. ferrooxidans* and *A. aeolicus* SqrAs) or as a stick and ball (in TrSqrF)

Three microbial SQRs (type I enzymes of *Acidithiobacillus ferrooxidans* and *Aquifex aeolicus* (Marcia et al. 2009; Cherney et al. 2010; Cherney et al. 2012), one type V enzyme from the hyperthermophilic archaebacterium *Acidianus ambivalens* (Brito et al. 2009), and only one eukaryotic SQR (human type II) (Jackson et al. 2019) have been structurally characterized thus far. SqrA and human SqrB proteins were crystallized in their decylubiquinone (dUQ)-bound forms. According to the crystal structures of SQR-dUQ complexes, the quinone binding site (QBS) of SQR enzymes is a channel in the C-terminal domain that connects the protein surface to the active center’s FAD cofactor. The apolar side-chain amino acids Val354, Phe357, and Phe394 for *A. ferrooxidans* SQR and Ile346, Ile348, and Phe385 for *A. aeolicus* SQR are located immediately adjacent to the redox headgroup of the dUQ molecule associated with the enzyme (PDB ID: 3T31 and 3HYW, respectively). When compared spatially, these residues form very similar channel structures in the two enzymes (Fig. 2). The residues *Af*Val355 and *Af*Phe357, as well as *Aa*Ile346 and *Ae*Ile348, are located on both sides of a conserved cysteine in the C-terminal domain’s first β-sheet element. As a result, their apolar side chains are adjacent. The *Af*Phe394 and *Aa*Phe385 residues are located in the first α-helix of these SqrA enzymes’ C-terminal domains (Fig. 2). The QBS of the *A. ambivalens* SqrE could not be determined because the C-terminal region of the crystallized proteins is structurally disordered, making it impossible to resolve its structure in this region (Brito et al. 2009; Marcia et al. 2009). Based on the homology model of the archaeal type III SqrC enzyme from *Caldivirga maquilingensis*, the Phe337 and Phe362 residues in this protein were identified as corresponding to the *A. ferrooxidans* SqrA Phe357 and Phe394 residues (Lencina et al. 2020). The SQR structures revealed that the dUQ molecule’s polar benzoquinone headgroup is sandwiched between branched side chain apolar amino acids and phenylalanine, or two phenylalanine benzene rings. The interactions between the amino acids that form the channel’s apolar walls and the quinone headgroup also provide the proper structure for electron transfer between the FAD and quinone molecules (Brito et al. 2009; Cherney et al. 2010). Additionally, the strongly hydrophobic decyl-chain of dUQ interacts with the side chains of several non-conserved amino acids located at the quinone channel’s entrance (Tyr323, Asn353, Ile368, and Tyr411 in the *A. ferrooxidans* SqrA protein) (Cherney et al. 2010; Zhang et al. 2016).

In the case of the *A. ferrooxidans* SqrA and *C. maquilingensis* SqrC, site-directed mutagenesis was used to disclose the role of amino acids located near the redox head moiety of the bound quinone molecule in the formation of QBS and the catalytic process of quinone reduction. Replacement of *Af*Phe357 and/or *Af*Phe394 residues with alanine resulted in a significant decrease and elimination of *A. ferrooxidans* SqrA activity; however, these amino acid substitutions had no effect on the enzyme’s affinity for dUQ (Zhang et al. 2016). Similar effects were observed for the activity and kinetic parameters of the *C. maquilingensis* type III SQR Phe362Ala mutant (Lencina et al. 2020).

*Thiocapsa roseopersicina* BBS (Bogorov 1974) is a model organism for photosynthetic purple sulfur bacteria because it possesses a complex sulfur metabolism capable of oxidizing various inorganic reduced sulfur compounds (e.g., sulfide) (Visscher et al. 1990; Tengölics et al. 2014). Both type IV and type VI SQR enzymes (TrSqrD and TrSqrF, respectively) were identified in *T. roseopersicina*, characteristic of purple sulfur bacteria (Duzs et al. 2018). The difficulty of expressing SQR enzymes in heterologous hosts and purifying them in an active form limits their structural, biochemical, and functional studies. However, using a genetic system developed for this strain (Fodor et al. 2001), the TrSqrF enzyme could be produced by their natural host cells in recombinant, mutant forms). Our previous study of the *T. roseopersicina* SqrF gene expression and kinetic analysis of homologously expressed and purified TrSqrF revealed that type VI SQR enzymes are responsible for sulfide oxidation at high concentrations (Duzs et al. 2018; Shuman and Hanson 2016; Chan et al. 2009). By analyzing single-cysteine mutant TrSqrF variants, we investigated the role of cysteine residues located in an unusual spatial structural pattern in type VI SQR in *T. roseoperscina* BBS. Based on these studies, a novel catalytic process for sulfide oxidation was proposed for type VI SQR enzymes (Duzs et al. 2021).

In this study, we examined the quinone reduction phase of type VI SQR-catalyzed redox reactions. The TrSqrF structure–function analysis was performed using molecular modeling and site-directed mutagenesis to deduce the molecular events occurring at the QBS of the model type VI SQR enzyme.

## Materials and methods

### Bacterial strains, plasmids, primers, nucleotide sequences

Bacterial strains, plasmids, and PCR primers used in this study are listed in Tables S1, S2, and S3. The *sqrF* gene sequence of *T. roseopersicina* is available in GenBank under the accession number KY595105.

### Growth conditions

*Escherichia coli* and *T. roseopersicina* cells were grown according to the protocol described in Duzs et al. 2018. *E. coli* cells were grown at 37 °C in Luria–Bertani (LB) medium supplemented with either kanamycin (25 µg mL^−1^) or ampicillin (100 µg mL^−1^). *T. roseopersicina* strains were grown for 4 days under anaerobic photoautotrophic conditions at 25 °C in a modified Pfennig’s medium containing 4 g L^−1^ sodium thiosulfate (Pfennig 1961). In some cases, the medium was supplemented with 25 µg mL^−1^ streptomycin, 25 µg mL^−1^ gentamycin, and 25 µg mL^−1^ kanamycin.

### Construction of expression vectors for production of TrSqrF mutant enzymes

Various nucleotide substitutions were introduced into the *sqrF* gene of *T. roseopersicina* using the QuickChange site-directed mutagenesis method (Stratagene) and the pBSQNNS cloning plasmid harboring the wild-type (WT) *sqrF* gene fragment as a template (Duzs et al. 2018). In a 50-µL total volume, an in vitro DNA synthesis reaction mixture contained 20 ng template plasmid, 1 × Phusion HF buffer, 200 µM dNTP, and 125–125 ng mutagenic primers (onV331AF-onV331AR, onV331FF-onV331FR, onI333AF-onI333AR, onI333FF-onI333FR, onF366AF-onF366AR); 2.5 U Phusion DNA polymerase (Thermo Fisher Scientific) was added to the reaction, and the following thermal profile program was applied: 1 × (30 s at 95 °C), 16 × (30 s at 95 °C followed by 1 min at 55 °C), and the synthesis was completed with a 6-min incubation at 72 °C for 6 min. After the DNA synthesis, the reaction mixture was supplemented with *Dpn*I (1 U) and incubated at 37 °C for 1 h before transforming the product into *E. coli* Novablue cells (Novagene/Merck & Co. Inc.). In order to confirm the mutations, plasmids were purified from selected clones and sequenced.

Vectors containing the mutant *sqrF* gene fragments (pBSQNNSV331A; pBSQNNSV331F; pBSQNNSI333A; pBSQNNSI333F; pBSQNNSF366A; pBSQNNSF366Y; pBSQNNSCTD) were cleaved by *Hin*dIII, and the ends were polished by Klenow enzyme and cleaved by *Nde*I endonuclease. The pDSK6CrtKm expression vector was digested by *Pst*I and blunted using T4 DNA polymerase, digested by *Nde*I and dephosphorylated using calf intestinal alkaline phosphatase. The DNA fragments of the mutant *sqrF* genes were inserted into the pDSK6CrtKm vector, yielding the pDSQNNSV331A, pDSQNNSV331F, pDSQNNSI333A, pDSQNNSI333F, pDSQNNSF366A, pDSQNNSF366Y, and pDSQNNSCTD expression vectors. Following sequence verification, the expression vectors were conjugated into the *T. roseopersicina* FOQRON strain described in Duzs et al. (2021) using the *E. coli* S17-1 lambda *pir* cell line (Fodor et al. 2001). The strains with the correct vectors were designated TrV331A, TrV331F, TrI333A, TrI333F, TrF366A, TrF366Y, and TrCTD strains (Table S1).

### Expression and purification of TrSqrF variants

*T. roseopersicina* cells expressing the mutant TrSqrF enzyme were grown for 4 days at 25 °C in 2 L modified Pfennig’s medium in Erlenmeyer flasks with a ground glass joint. The cultures were centrifuged at 8,300 × g at 4 °C for 10 min. To prepare spheroplasts, harvested cells were suspended in periplasmic buffer (TBS buffer pH = 8.0 (150 mM NaCl, 50 mM Tris–HCl), 25% sucrose, and 0.1% lysozyme) and incubated at 30 °C for 30 min. The suspensions were then added to an equal volume of ice-cold ion-free water and incubated on ice for an additional 10 min. The cell suspensions were then centrifuged (13,700 × g, 4 °C, 15 min), and the spheroplasts were suspended in TBS buffer and sonicated eight times for 15 s with 7 kJ total energy (Bandelin Sonoplus HD3100) with continuous ice-cooling, followed by centrifugation (27,000 × g, 4 °C, 15 min) to separate the cell debris. The supernatant obtained comprises crude cell extracts, which was ultra-centrifuged (100,000 × g, 90 min, 4 °C) (Sorwall WX Ultra Series). The pellet (membrane fraction) was suspended in TBS buffer, supplemented with 10 µM EDTA (pH = 8.0) and incubated at room temperature for 30 min with continuous shaking. Sodium bromide was then added to 1.45 M final concentration, and incubation continued for an additional 60 min at 25 °C. After ultracentrifugation (200,000 × g, 120 min, 4 °C) of the treated membrane fraction sample, the supernatant containing the solubilized TrSqrF mutant protein was collected and stored at - 20 °C. Purification of recombinant mutant TrSqrF proteins containing an N-terminally fused StrepII tag peptide was performed at room temperature using Strep-Tactin Superflow High-Capacity resin (IBA Life Sciences, cat. no.: 2–1208-002) following the instructions of the manufacturer.

### Protein analytical methods

The purified TrSqrF mutants were analyzed on 12% denaturing SDS polyacrylamide gels (Wittig and Schägger 2005). Proteins (1 µg) were loaded onto the gels, and — after electrophoresis — the protein bands were visualized using Blue Silver dye staining (Candiano et al. 2004). The recombinant TrSqrF fusion proteins were identified by western blot experiments using StrepII tag–specific HRP conjugated monoclonal antibodies (IBA Lifesciences). Subsequently, the SuperSignal West Pico Rabbit IgG Detection Kit (Thermo Scientific) was used to generate chemiluminescence, and the VersaDoc 4000 MP gel-imaging system was used to detect signals (BIO-RAD). For determination of molecular weight of the purified TrSqrF mutant proteins, Prosieve Quadcolor Protein Marker (catalog no. 00193837) was used. Protein concentrations were determined using the Lowry method (Lowry et al. 1951).

### Spectroscopic assays

The UV–visible absorption spectra of purified proteins were determined using a quartz cuvette and a spectrophotometer (Nicolet Evolution 300, Thermo Fisher Scientific). The absorbance values were measured in the range of 300–700 nm. Due to the variable concentrations of the protein samples, absorption spectra of the purified TrSqrF variants were normalized to the same maximal absorption values.

### Enzyme activity assays and kinetic parameters

Sulfide-dependent quinone reduction activity of TrSqrF mutants was measured in nitrogen-flushed quartz cuvettes sealed with Suba-seal septa (Sigma-Aldrich) under anaerobic conditions. The standard reaction mixtures contained 50 mM Tris–HCl pH = 8.0 buffer, 50 µM duroquinone (DQ) or dUQ, and 6.6 µg (for DQ) or 1 µg (for dUQ) purified enzyme in 1 mL final volume. The reduction of DQ (molar extinction coefficient at 275 nm: 16 mM^−1^ cm^−1^ (Degli Esposti et al. 1982)) and dUQ (molar extinction coefficient at 278 nm: 12.6 mM^−1^ cm^−1^ (Kröger 1978)) was monitored at 275 and 278 nm, respectively, at 25 °C using a spectrophotometer (Nicolet Evolution 300, Thermo Scientific). In order to initiate enzyme reactions, 200 µM freshly prepared anaerobic sulfide solution was added to the reaction mix. The kinetic constants were calculated using curves plotting enzyme activity against various concentrations of DQ (5–150 µM) and dUQ (5–100 µM). The curves were fitted data non-linear regression analysis of MatLab software (Dorf and Bishop 2011). A unit of activity of the sulfide/quinone oxidoreductase was defined as the reduction of 1 µmol quinone per min.

### In silico analysis of protein–ligand interactions

The initial structure of the TrSqrF molecular dynamics (MD) calculations was the homology model previously described (Duzs et al. 2021), in which the type VI SQR enzyme associated with dUQ and FAD cofactors was determined based on the crystal structures of *A. ambivalens* SqrE (Brito et al. 2009) and *A. aeolicus* SqrA (Marcia et al. 2010b). MD calculations were performed using the Desmond package (Schrödinger Release 2021–1) (Bowers et al. 2006) of the Schrödinger software suites applying the OPLSA2005 (Jorgensen et al. 1996) force field in combination with the SPC explicit water model. The dUQ was in its keto form, and the FAD was covalently attached to the protein via Cys121. A 250-ns long Replica Exchange Solute Tempering (REST) calculation (Wang et al. 2011) was used to ensure that the dUQ ligand and its 10 Å protein environment were properly sampled. The REST region selected contained the dUQ ligand and its 10 Å protein environment, as well as the FAD cofactor. During the 250-ns simulation, the first 50-ns part of the trajectory can be considered as the relaxation of the system. The temperature range was determined automatically by the Schrödinger program, where the lowest replica was at 300 K. The Simulation Interaction Diagram protocol from the Schrödinger package was used to examine the interaction pattern of the dUQ ligand for the trajectory at 300 K.

## Results

### Sequence comparison-based identification of the QBS of TrSqrF

For identification of the amino acids likely forming a potential quinone binding pocket in TrSqrF, the C-terminal domains of various types of SQRs were aligned (Fig. 1). The Val331, Ile333, and Phe366 residues in TrSqrF are in the corresponding sequence positions as the amino acids forming quinone channel in SqrA enzymes. To analyze the spatial distribution of amino acids in TrSqrF, we used an energy-minimized homology structural model of the enzyme (Duzs et al. 2021). Based on this model structure, the Val331, Ile333, and Phe366 residues in this protein can form a channel. A comparison of the SQR structures revealed that the TrSqrF residues have the same spatial arrangement as the corresponding amino acids in the SqrA enzymes (Fig. 2). The redox head group of the quinone is located between the side chain of Val331 and the aromatic ring of Phe366, similar to the *A. aeolicus* SqrA, where the redox group of the dUQ is located between *Aa*Ile346 and *Aa*Phe385 (Marcia et al. 2009). In contrast, the benzoquinone ring of bound dUQ is sandwiched between *Af*Phe357 and *Af*Phe394 residues in the crystal structure of *A. ferrooxidans* SqrA protein (Cherney et al. 2010).

### In silico analysis of the interactions between the TrSqrF and dUQ

**Fig. 3:**
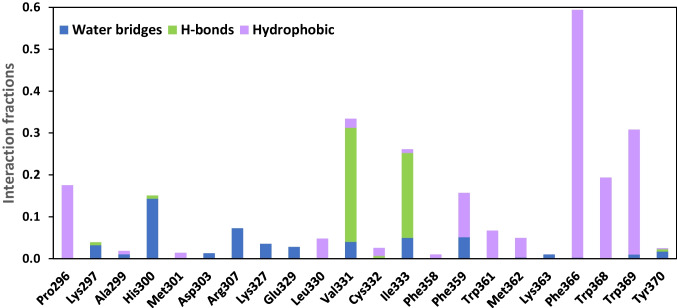
Significant secondary interactions between the dUQ ligand and the TrSqrF protein

**Fig. 4:**
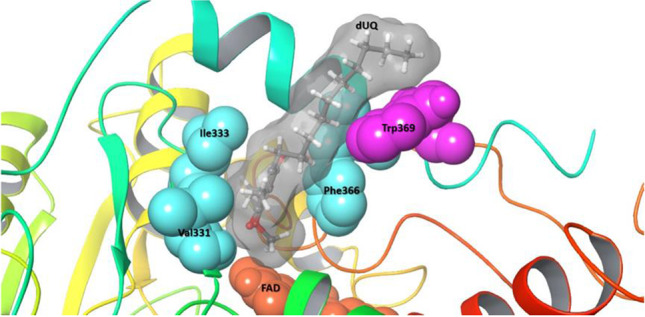
Representative structure of the TrSqrF–decylubiquinone (dUQ) complex of *T. roseopersicina*. In surface representation, the apolar amino acids Val331, Ile333, and Phe 366 that comprise the quinone binding site (QBS) are labeled (colored in cyan). Bound dUQ is denoted by stick and ball marks, and the molecule’s surface is also shown (colored in gray). Trp369 (magenta) interacted with the ligand’s decyl side chain. Additionally, the flavin adenine dinucleotide (FAD) cofactor is depicted as a surface representation (colored in orange)

The protein–ligand interactions were modeled to gain a better understanding of the role of the TrSqrF residues identified in quinone binding. The REST method was used to obtain a molecular-level picture of the stability of the dUQ–protein interaction (see “Materials and methods” and “In silico analysis of protein–ligand interactions”). We discovered that the dUQ remained in its binding pocket throughout the entire simulation at each replica, indicating that our model accurately represents a stable structure. When the replica with the lowest temperature was analyzed, the root mean square deviation (RMSD) values of the ligand’s heavy atom typically ranged between 3 and 6 Å during the 250-ns simulation, with the exception of the first 50 ns (Fig. S1). However, this part of the trajectory can be viewed as the relaxation of the system. Additionally, a significant portion of the mobility is due to the flexibility of the hydrocarbon tail part of the dUQ (Fig. S2), which likely contributes to the relative stability of the position of the head region. The interaction between the dUQ ligand and the protein observed in the simulated structures of TrSqrF − dUQ complexes is summarized in Fig. 3 which shows that only a few secondary interactions keep the ligand in the binding pocket. The quinone head group appears to interact most frequently with the Val331, Ile333, and Phe366 residues, as well as with the tail of the dUQ molecule and Trp369 (Fig. S3). The dUQ binds to the hydrophobic Val331 or Ile333 via H-bridges that can be formed between the backbone heteroatoms of these amino acids and the oxo-groups of the head part of the dUQ ligand (Fig. 3). The distances between the hydrogen of the peptide amide groups of Val331 and Ile333 residues and the O4 and O1 atoms of dUQ are 2.3 ± 0.2 Å and 2.3 ± 0.4 Å, respectively. These are sufficiently short to allow for the formation of H-bridges. The dUQ-Phe366 connection appears to be the most significant hydrophobic interaction. The center of dUQ’s benzoquinone ring is located at 4.2 ± 0.3 Å from the center of the benzene ring of Phe366. Moreover, the benzoquinone ring is structurally similar to the isoalloxazine group of the FAD. When the O_2_ atom of FAD and the O_4_ atom of dUQ are in close proximity, the distance between them is 5.3 ± 0.3 Å in the simulated structures. The representative structure of the TrSqrF-dUQ-FAD is shown in Fig. 4. Based on the sequence and structural alignments, as well as predicted chemical interactions between the dUQ head group and the protein, the Val331, Ile333, and Phe366 residues were further analyzed biochemically to get deeper insight into their roles in quinone binding and catalytic activity of TrSqrF.

### Expression and purification of QBS mutant TrSqrF variants

In order to further characterize the role of selected amino acids in quinone binding and catalysis, Val331, Ile333, and Phe366 mutant TrSqrF enzymes were created and biochemically characterized. The amino acids were replaced with alanine (V331A, I333A, F366A) and aromatic amino acids (V331F, I333F, F366Y) to demonstrate the importance of hydrophobic interactions between these residues and the quinone head group for enzyme activity. Additionally, to elucidate the function of the single α-helix segment of C-terminal domain of TrSqrF (which contains the Phe366 residue and thus contributes to the formation of the quinone pocket), a C-terminal α-helix deletion mutant enzyme variant (CTD) was constructed by inserting triple TGA stop codons after the nucleotides encoding the Thr356 residue in the *sqrF* gene.

**Fig. 5:**
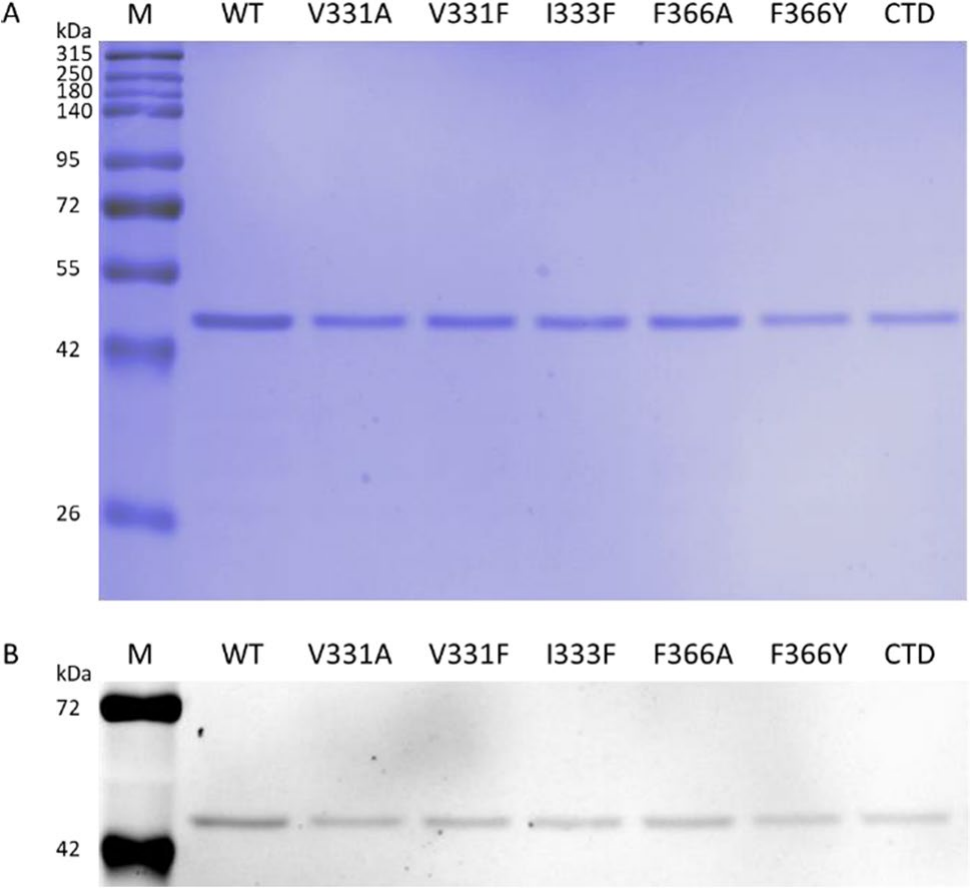
SDS-PAGE analysis of the purified wild-type (WT) and the TrSqrF QBS mutants. A total of 1 µg of proteins was loaded. The first lanes were loaded with a standard molecular weight marker (M). **A** Blue Silver staining of SDS-PAGE gel. **B** The same SDS-PAGE gel, but unstained, was illuminated with UV light to detect the fluorescent signals of FAD

The wild-type and a few StrepII affinity tag–fused TrSqrF variants were expressed in a *T. roseopersicina* strain (FOQRON) lacking sulfide oxidase enzymes (TrSqrD, TrSqrF, and FccAB) (Duzs et al. 2018, 2021). According to Duzs et al. 2018, TrSqrF variants carrying mutations in the quinone binding pocket were generated. Similarly to previous publications, the membrane fraction of *T. roseopersicina* cells contained recombinant TrSqrF point mutants (data not shown). The membrane binding of the CTD variant (Fig. S4) was unexpected, given the previously reported critical role of the C-terminal α-helix in *C. maquilingensis* SqrC membrane attachment (Lencina et al. 2013). Purification of wild-type and mutant TrSqrFs from solubilized membranes was accomplished using affinity chromatography. The yields of the point mutants (45.5 ± 24.9 µg/L) were comparable, except for the I333A mutant, which could not be purified despite its presence in the cell membrane fractions. Surprisingly, the purification yield of the CTD mutant (320 ± 77.8 µg/L) was significantly greater than that of the wild-type TrSqrF (48.2 ± 7.7 µg/L). SDS-PAGE analysis revealed a single band with a molecular weight of approximately 44 kDa in each sample, indicating that the purified TrSqrF mutants were pure and homogeneous (Fig. 5).

### Spectroscopic analysis of quinone biding site mutant TrSqrF proteins

**Fig. 6:**
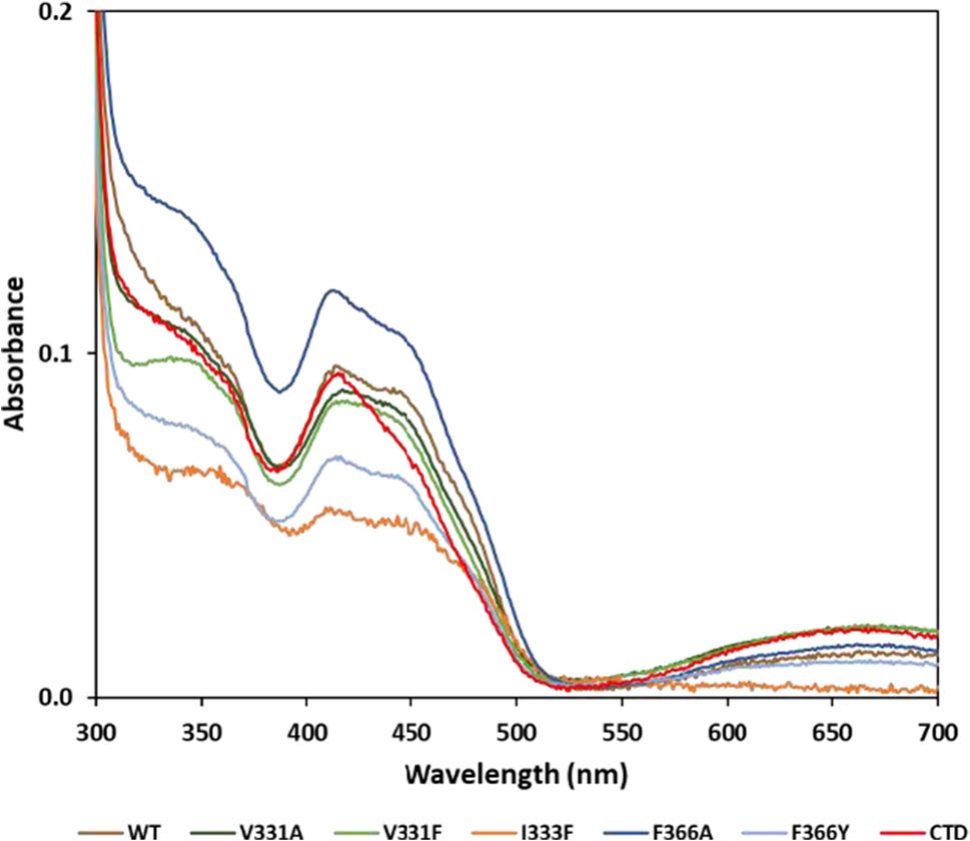
UV–visible absorption spectra of the purified WT (colored in brown), V331A (dark green), V331F (light green), I333F (orange), F366A (dark blue), F366Y (light blue) QBS point mutants and C-terminal α-helix deletion mutant (colored in red) TrSqrF variants (8.2 µM, 25.7 µM, 31.9 µM, 9.5 µM, 9.1 µM, 14.8 µM, and 11.4 µM, respectively). The absorption spectra of the protein samples were normalized to the absorption value at 280 nm

The absorption spectra of pure TrSqrF variants were recorded to verify the cofactor content and redox state of the SQR enzymes (Fig. 6). The oxidized forms of the tDBDF enzyme family, including SQRs, have characteristic absorption peaks at 360 and 448 nm (Brito et al. 2009). Absorption maxima at these wavelengths indicated that all TrSqrF variants contained the FAD cofactor (Fig. 6). The FAD content of mutant proteins was calculated using an absorption coefficient of 11.3 mM^−1^ cm^−1^ (Dawson 1989). In most instances, the FAD-protein ratio was approximately 0.62, which was consistent with the WT enzyme (Fig. 5B) (Duzs et al. 2018). According to the absorption spectrum, the I333F mutant contained a slightly lower concentration of FAD (0.43). The FAD content of the enzyme variants was also confirmed by the fluorescence emission of protein bands in an SDS-PAGE gel illuminated with UV light (Fig. 5B). Except for the I333F mutant, a broad absorption band with a maximum at 675 nm is also visible in the spectra of the TrSqrF variants (Fig. 6). This band indicates the presence of a charge transfer complex (CTC) formed between the FAD cofactor and a thiolate group during the catalytic cycle of SQR enzymes (Griesbeck et al. 2002; Mishanina et al. 2015) and type II NADH/quinone oxidoreductases (Sousa et al. 2017; Sena et al. 2018). The similar spectra in the 400–500-nm wavelength range show that the FAD cofactor is in corresponding oxidized state in the purified single amino acid mutants. In contrast, the CTD mutant has a lower absorbance at 448 nm, indicating a higher proportion of reduced proteins (Fig. 6). In order to estimate the redox state of the purified CTD mutant, the protein was reduced by sodium-dithionite and re-oxidized by DQ. After the addition of DQ, the absorption peak of comparable height at 448 nm reappeared in the spectrum of the CTD mutant (Fig. S5). Likewise, previously published TrSqrF variants (Duzs et al. 2021), a weak additional absorbance peak at 408 nm was observed in the UV–Vis spectra of these TrSqrF mutants, indicating the presence of trace amounts of c-type cytochromes in the samples (the extinction coefficient of heme-containing c-type cytochromes at 408 nm is high (170 mM^−1^ cm^−1^) (Sousa et al. 2017; Sena et al. 2018). The amount of c-type cytochrome contamination in SDS-PAGE is less than the detection limit (Fig. 5).

### Activity and kinetic parameters of QBS mutant TrSqrF variants

**Fig. 7:**
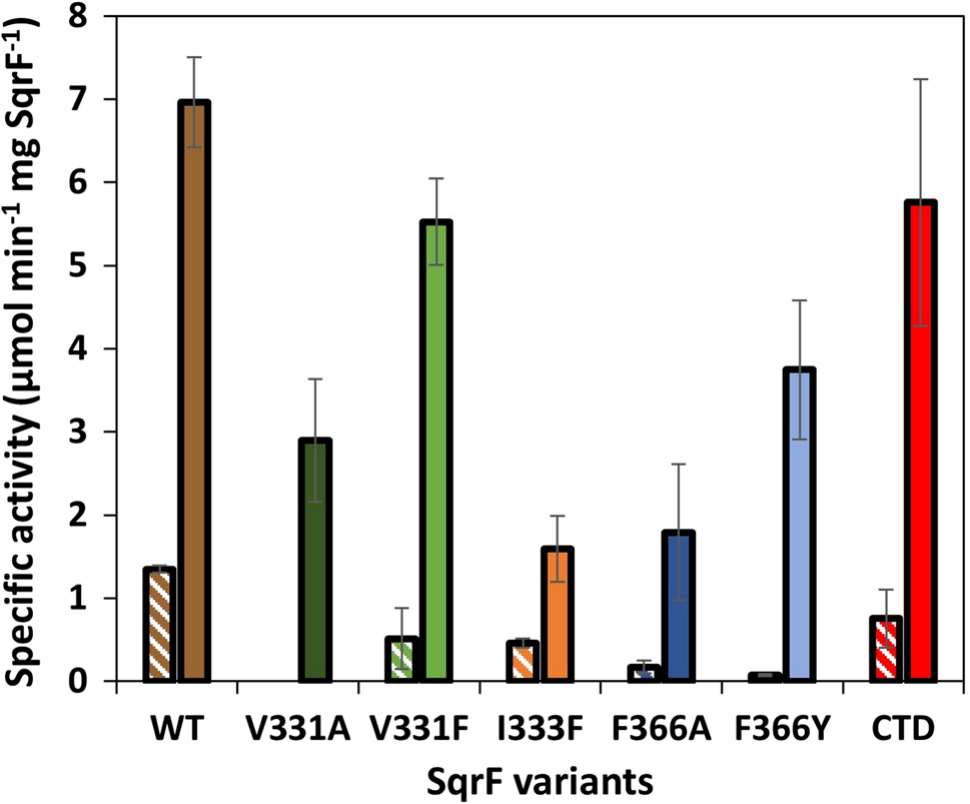
Sulfide-dependent DQ (striped columns) and dUQ (solid-colored columns) reducing specific activity of the purified WT and QBS mutant TrSqrF enzymes

In order to disclose the functional role of the amino acids forming QBS during the catalysis by TrSqrF, sulfide-dependent quinone reducing activity of the enzyme variants was measured. Our previous studies revealed that TrSqrF prefers ubiquinone-type quinones such as DQ and especially dUQ (Duzs et al. 2018). In order to conduct a comparative analysis of the catalytic properties of the TrSqrF variants, both DQ and dUQ reductase activities were measured by standard activity assay (see “Materials and methods” 7.2) (Fig. 7). All TrSqrF variants catalyzed the DQ or dUQ reduction with significantly lower specific activity than the WT enzyme. The V331A mutant was completely incapable of reducing DQ. The substitution of phenylalanine for Val331 and Ile333 resulted in a decrease in DQ reducing activity, whereas mutations of the Phe366 residue (F366A, F366Y) resulted in the greatest decline in DQ-reducing activities, which are now only 13% and 6% of WT TrSqrF activity, respectively.

Substituting aromatic phenylalanine (V331F) for Val331 resulted in a significantly smaller decrease in activity than the valine-to-alanine mutation. The I333F mutant had the lowest specific activity with dUQ of the TrSqrF variants. In the case of the Phe366 residue, its replacement by an aromatic amino acid (F366Y) had a smaller effect on dUQ reduction than the substitution with alanine, which contrasted with their relative effect on DQ-reducing activity. Interestingly, deletion of the C-terminal α-helix had a smaller effect on the enzyme’s activity than the single α-helix point mutations (F366A, F366Y). The specific activities of TrSqrF variants measured in the standard activity conditions were highly consistent with the *k*_cat_ parameters of the enzymes determined via steady-state kinetic measurements (Table 1).

**Table 1:** Steady-state kinetic parameters of wild-type and QBS mutant TrSqrF enzymes for DQ and dUQ substrates

Protein	*k*_cat_ (s^−1^)		*K*_*m*_ (μM)		*k*_cat_/*K*_*m*_ (μM^−1^ s^−1^)	
	DQ	dUQ	DQ	dUQ	DQ	dUQ
SqrF WT	1.25 ± 0.04	8.37 ± 0.33	27.39 ± 2.47	24.47 ± 6.42	0.046	0.342
SqrF V331A	0	2.47 ± 0.33**	n.d	18.99 ± 6.10	n.d	0.130
SqrF V331F	0.72 ± 0.02**	3.64 ± 1.06**	40.23 ± 7.36*	12.33 ± 0.86*	0.018	0.295
SqrF I333F	0.72 ± 0.21*	1.20 ± 0.48**	102.7 ± 69.9	13.98 ± 7.20	0.007	0.086
SqrF F366A	0.26 ± 0.01**	1.97 ± 0.28**	46.19 ± 2.53**	9.38 ± 1.97*	0.006	0.210
SqrF F366Y	0.57 ± 0.08**	3.17 ± 0.82**	79.30 ± 14.68**	10.03 ± 1.39*	0.007	0.316
SqrF CTD	0.96 ± 0.24	4.90 ± 0.80**	70.71 ± 44.78	18.99 ± 0.67	0.014	0.268

The Welch’s *t* test was used to calculate the significance values in R Studio software

^*^p-value < 0.05; **p-value < 0.005

For both types of ubiquinone, the steady-state kinetic parameters of TrSqrF variants were determined. After determining the mutants’ kinetic curves for quinone substrates, the *k*_cat_ and *K*_*m*_ parameters, as well as the efficiency values (*k*_cat_/*K*_*m*_) of their quinone reducing activities, were calculated applying non-linear regression fitting to the Michaelis–Menten equation. The increased *K*_*m*_ values indicated that all mutations decreased the enzyme’s affinity for DQ in comparison to the WT enzyme (Table 1). However, none of the mutants had a decreased affinity for dUQ. The V331A, I333F, and CTD variants all had comparable or slightly lower *K*_*m*_ values for this substrate than WT TrSqrF. The V331F, F366A, and F366Y mutants had lower *K*_*m*_ values, indicating that these variants have a higher affinity for dUQ (Table 1).

**Fig. 8:**
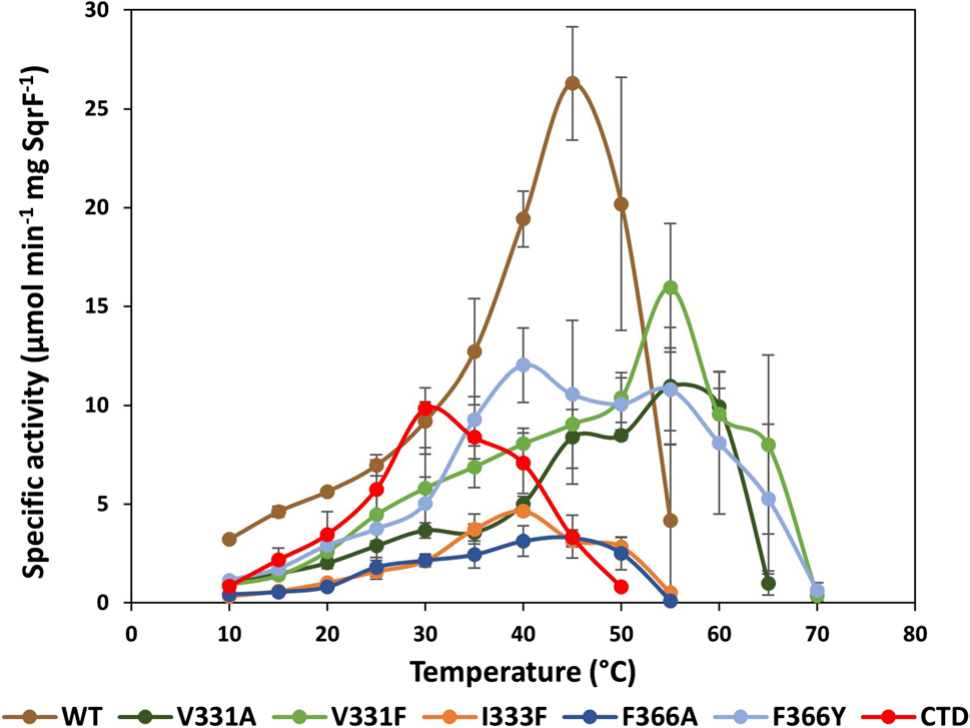
Temperature dependence of dUQ reducing activity of wild-type and TrSqrF QBS mutants, with the color key provided

In Fig. 8, the temperature profiles of the mutant enzymes are compared to those of the WT TrSqrF, which has a maximum activity temperature of 45 °C. Each modification in the TrSqrF sequence decreased the maximal activity of the enzyme variants. As Fig. 8 shows, the optimal temperatures of the Val331 variants (V331A, V331F) are slightly higher (55 °C), whereas those of the Ile333 and Phe366 mutants are slightly lower (40–45 °C) relative to WT. The CTD mutant exhibited the lowest temperature optimum (30 °C).

The thermostability of the CTD mutant (with the lowest optimal temperature) was also determined in the following step by monitoring the activity of enzyme samples that had previously been treated at various temperatures (50–65 °C) for varying times (0–60 min). The CTD mutant and WT enzyme had comparable thermostability, indicating that the deletion of the C-terminal α-helix had no destabilizing effect on the enzyme structure (Fig. S6).

## Discussion

The mechanism of electron transfer from FAD to quinone in SQRs and the structure–function relationship of the quinone channel is poorly understood. This could be due to the heterogeneity of the quinone binding motifs of various enzymes despite their overall structural similarity. Quinones are bound to the C-terminal domain of SQRs near the FAD. The C-terminal regions of SQRs vary in terms of the number of amphipathic α-helices and amino acids involved. The multiple sequence alignment (Fig. 1) indicates that the quinone binding pocket is characterized by highly conserved apolar amino acids. One side of the channel comprised aromatic amino acids, and the opposite side is usually formed by branched side chain apolar amino acids.

Our primary objective was to decipher the structure–function relationship of the QBS in a novel type VI SQR isolated from *T. roseopersicina*. A homology model for the WT enzyme was constructed (Duzs et al. 2021) using previously crystallized SQRs (*A. ferrooxidans*, *A. aeolicus* SqrA, and *A. ambivalens* SqrE) (Brito et al. 2009; Marcia et al. 2009; Cherney et al. 2010). The apolar amino acids phenylalanine, valine, and isoleucine at the C-terminus of the protein were identified as putative residues for the TrSqrF QBS based on primary sequence comparisons and a structural model (Fig. 4). Other type I SQRs, such as *A. ferrooxidans* and *A. aeolicus*, contain similar apolar residues (Marcia et al. 2009; Cherney et al. 2010, 2012). Nevertheless, conserved amino acids of the same type may differ in putative QBSs (Fig. 1).

In silico structural modeling and in silico protein–ligand interaction analysis revealed that four amino acids (Val331, Ile333, Phe366, and Trp369) may play a significant role in the binding and orientation of the dUQ ligand (Fig. 4). According to the structural models, the Trp369 had a hydrophobic connection only with the decyl-chain of the dUQ. *Af*Tyr411 of *A. ferrooxidans* SqrA is in a similar position, close to the QBS channel’s entrance; interacts with the hydrophobic quinone tail; and is presumably responsible for protonation of the reduced quinone (Cherney et al. 2010; Zhang et al. 2016). Contact studies revealed the positions of the Val331, Ile333, and Phe366 residues near the dUQ’s benzoquinone ring, implying their putative role in quinone binding and dUQ’s positioning for electron transfer. The binding of dUQ to Val331 and Ile333 is unexpected, as both amino acids are mainly hydrophobic. The interaction graph (Fig. 3) disclosed that Val331 and Ile333 formed a hydrogen bond with the ligand. These small amino acids in the quinone binding pocket may interact with the dUQ oxo-groups O4 (Val331) and O1 (Ile333).

However, the interaction of dUQ with V331 and I333 alternated, and the Val331- and Ile333-associated with dUQ represented 33% and 26% of the potential structures, respectively (Fig. S3). On the other hand, the benzene ring of Phe366 formed a hydrophobic bond with the redox head group of quinone. Throughout the investigated intervallum, the Phe366-dUQ interaction predominated (approximately 60% of the potential structures) (Fig. S3). These amino acids appear to play a role in maintaining the quinone substrate’s optimal position. As dUQ could interact with the QBS via its long aliphatic side chain, it was less flexible than DQ. This is hypothesized to be a possible explanation for the increased activity observed with dUQ.

While the hydrophobic amino acids in the QBS are not conducive to electron transfer, the interactions described above ensured that the benzoquinone ring of dUQ remained near the isoalloxazine moiety of the FAD cofactor. Due to the close proximity of the two redox active molecules (4.3 Å), a direct electron transfer from the cofactor to the ligand may occur, similar to *A. ferrooxidans* SqrA, where the distance between the O4 atom of dUQ and the O2 atom of FAD is approximately 3 Å (Cherney et al. 2010). Between Val331 and Ile333 (Fig. 1), a cysteine residue (Cys332) may also be responsible for the proper geometric positioning of the FAD cofactor. This cysteine residue was involved in the transfer of the polysulfur chains of oxidized sulfides during the reductive phase (Duzs et al. 2021).

To conduct functional analysis, we used site-directed mutagenesis to generate and analyze alanine (V331A, I333A, F366A) and aromatic (V331F, I333F, F366Y) amino acid variants for each of three primary candidates for binding the benzoquinone in TrSqrF. Furthermore, a TrSqrF C-terminal α-helix deletion (CTD) mutant was constructed. Spectroscopic analysis confirmed the presence of the FAD cofactor in all mutants.

The kinetic data were determined using the substrates DQ and dUQ. DQ is a tetramethyl-p-benzoquinone without a tail, whereas dUQ contains methoxy and decyl groups on the p-benzoquinone ring. As a result, dUQ is asymmetric and contains a long linear hydrophobic chain. Although the dUQ is more similar to the proposed natural substrate of the TrSqrF, UQ8 (Duzs et al. 2018), DQ was also included in the measurements to reveal the effect of the linear aliphatic chain of dUQ on the enzyme-quinone interactions. The WT enzyme performed better with dUQ, which was more efficiently reduced (k_cat_/K_m_) than DQ (Duzs et al. 2018).

Substituting alanine for V331 and I333 resulted in either an unpurifiable (I333A) or a DQ-inactive (V331A) enzyme. The latter remained active in the presence of dUQ but had lower *k*_cat_, *K*_*m*_, and *k*_cat_/*K*_*m*_ values than the WT enzyme. In comparison, the V331F mutant has almost completely recovered its activity when treated with dUQ. Similarly to *A. ferrooxidans* SqrA, this case had two aromatic benzene rings on both sides of the QBS (Cherney et al. 2010). This arrangement was nearly identical to the QBSs characterized previously (Marcia et al. 2009). Notably, the Val331 TrSqrF mutants with higher optimal temperatures were found to be more thermophilic than the WT or other mutants. The substitution of Ile333 for Phe had no such beneficial effect. It significantly increased the *K*_*m*_ for DQ while maintaining affinity for dUQ comparable to that of other mutants. The band of the CTC complex, a transitional protein-FAD complex involved in electron transfer between the sulfide and the FAD cofactor, was observed in the spectra of all mutants except I333F. This could explain why the I333F TrSqrF mutant exhibited the lowest activity of all the enzymes examined. *Af*Phe357, *Aa*Ile348, and *Cm*Phe337 are located in the corresponding position of Ile333 in TrSqrF. With dUQ, the *Af*F357A mutant’s specific activity was approximately 10% of the specific activity of *A. ferrooxidans* SqrA (Zhang et al. 2016), whereas *Cm*F337A exhibited similar inactivity (Lencina et al. 2020).

Notably, while a Phe occurred in the position corresponding to TrSqrF’s Ile333 in *A. ferrooxidans* SqrA, this is not the case for all type I enzymes: Leu and Ile occurred in these positions in *R. capsulatus* and *A. aeolicus* SqrA, respectively (Fig. 1). Consequently, the amino acids responsible for binding the quinone ring via H-bonds should be small apolar residues such as Val, Leu, or Ile in types I, IV, V, and VI SQRs. Type IV enzymes are an exception to this rule, as a residue corresponding to Val331 Ala has been identified.

Replacement of Phe366 with alanine and tyrosine decreased the enzyme’s specific activity, particularly with DQ. This indicates that the quinone and FAD are also not in the proper spatial position for charge transfer in these mutants. Despite their decreased *K*_*m*_ for dUQ, these mutants exhibited decreased dUQ reducing activity, which may be a result of the altered spatial arrangement of the components of QBS. Phe occurred in the corresponding positions in all published studies to date (Marcia et al. 2009; Cherney et al. 2010). Nevertheless, other residues such as Thr, Tyr, and Trp have been identified at this site in a variety of SQRs. Additionally, substitutions of equivalent Phe residues in *A. ferrooxidans* SqrA (*Af*F394A) and *C. maquilingensis* SqrC (*Cm*F362A/Y/W) resulted in a significant decrease in activity (Zhang et al. 2016; Lencina et al. 2020).

Surprisingly, the CTD mutant was purified from the membrane fraction (Fig. 5) (Fig S4). The purification yield of CTD was unexpectedly high, approximately six-fold that of WT TrSqrF. As one of the presumed functions of the C-terminal region is membrane anchoring (Cherney et al. 2010), it was expected that removing the C-terminal α-helix would solubilize the protein more efficiently than the WT protein. The truncation of one or two C-terminal helix of *C. maquilingensis* SqrC resulted in a cytoplasmic protein without catalytic activity (Lencina et al. 2020). Similarly to *A. ambivalens* SqrE, the TrSqrF contains only one predicted α-helix, and this helix is shorter in type VI SQRs than in other SQRs (Marcia et al. 2010a) (Fig. 1). With DQ and dUQ, the CTD truncated TrSqrF retained 55% and 83% of the WT enzyme activity, respectively. Therefore, this mutant exhibited the highest activity for both quinones.

Given that Phe366 and Trp369 are located on the C-terminal α-helix of TrSqrF, it was surprising that substitutions for Phe366 resulted in a greater decrease in enzyme activity than the CTD mutant (Fig. 7). Nonetheless, the CTD TrSqrF mutant has the lowest temperature optimum (30 °C) among WT and mutant TrSqrF enzymes (Fig. 8). The thermostability of the CTD mutant was investigated, and it was discovered that removing the C-terminal end of the protein did not destabilize the enzyme (Fig. S6). One could hypothesize that the absence of the C-terminal helix results in a larger quinone binding pocket between the protein surface and the FAD in the active center, thereby altering the hydrophobicity. According to our hypothesis, dUQ may be able to enter the QBS easily and get close to the cofactor, but the electron transfer and stability of the enzyme–ligand complex are impaired.

In conclusion, this study demonstrates that during the oxidative stage of type VI SQR’s catalytic process, primarily Val331, Ile333, and Phe366 residues participate in the formation of the quinone binding channel and interact with the redox head group of quinones in the TrSqrF. By keeping the quinone’s benzoquinone ring in proper position, these amino acids have structural role in the catalytic electron transfer from FAD to quinone. In addition, Ile333 is involved in the formation of the charge transfer complex of FAD, while Trp369 interacts with the strongly hydrophobic quinone tail group outside the quinone pocket. The enzyme lacking the C-terminal α-helix remains membrane-anchored and active; however, this α-helix has important contribution to appropriate quinone binding.

## Supplementary Information

Below is the link to the electronic supplementary material.

Supplementary file1 (DOCX 1.67 MB)

## Data Availability

All data generated or analyzed during this study are included in this published article (and its supplementary information files).
