# Supplementary material for: Quinone binding site in a type VI sulfide:quinone oxidoreductase
Source: Appl Microbiol Biotechnol. 2022 Oct 11;106(22):7505–17. doi: 10.1007/s00253-022-12202-8 (PMC9666304; doi:10.1007/s00253-022-12202-8)
Supplement: Supplementary file 1 — Supplementary file1 (DOCX 1.67 MB) [file 253_2022_12202_MOESM1_ESM.docx]

**SUPPLEMENTARY MATERIAL**

**Quinone-binding site in a type VI sulfide:quinone oxidoreductase**

**Nikolett Miklovics^1,2,3^, Ágnes Duzs^1,2^, Fanni Balogh^1,2^, Gábor Paragi^4,5^, Gábor Rákhely^1,2,+,*^, András Tóth^1,2,+^**

^1^ Institute of Biophysics, Biological Research Centre, Temesvári krt 62., H-6726, Szeged, Hungary

^2^ Department of Biotechnology, University of Szeged, Közép fasor 52., H-6726, Szeged, Hungary

^3^ Doctoral School in Biology, University of Szeged, Közép fasor 52., H-6726, Szeged, Hungary

^4^ Institute of Physics, University of Pécs, Ifjúság útja 6., H-7624, Pécs, Hungary

^5^ MTA-SZTE Biomimetic Systems Research Group, Department of Medical Chemistry, University of Szeged, Dóm square 8, H-6720, Szeged, Hungary

*Corresponding author. Telephone: +36-30-3824172. Fax: +36 62-544352 e-mail: rakhely.gabor@brc.hu

^+^ These Authors equally contributed to the paper.

**Running title**

Quinone-binding site in a type VI sulfide:quinone oxidoreductase

**Keywords:** sulfide:quinone oxidoreductase (SQR); disulfide reductase, sulfur metabolism, quinone binding, quinone reduction

**Table S1 Bacterial strains used in the study.**

| Bacterial strain | Genotype | Reference |
| --- | --- | --- |
| *Escherichia coli* |  |  |
| XL-1 Blue MRF’ | *recA1 endA1 gyrA96 thi-1 hsdR17 supE44 relA1 lac* [*F´ proAB* *lacI*^q^  Z∆*M15* Tn*10* (Tet^r^)] | Stratagene |
| S17-1 lambda pir | 294 (*recA pro res mod*) Tp^r^ Sm^r^ (pRP4-2-Tc::Mu-Km::Tn*7*) *λpir* | (Palágyi-Mészáros 2006) |
| *Thiocapsa roseopersicina* |  |  |
| FOQRON | *HynSL*::Sm^r^ *hupSL*::Gm^r^ *ΔfccΔsqrDΔsqrF* | (Duzs et al. 2021) |
| TrV331A | FOQRON + pDSQNNSV331A | This work |
| TrV331F | FOQRON + pDSQNNSV331F | This work |
| TrI333A | FOQRON + pDSQNNSI333A | This work |
| TrI333F | FOQRON + pDSQNNSI333F | This work |
| TrF366A | FOQRON + pDSQNNSF366A | This work |
| TrF366Y | FOQRON + pDSQNNSF366Y | This work |
| TrCTD | FOQRON + pDSQNNSCTD | This work |

**Table S2 Plasmids used in the study.**

| Name | Feature | Reference |
| --- | --- | --- |
| pBluescriptSK + | ColE1, cloning vector, Amp^r^ | Stratagene |
| pDSK6CrtKm | pDSK509 based expression vector with promoter of *T. roseopersicina crtD* gene, Km^r^ | (Balogh et al., personal) |
| pBSQNNS | pBluescriptSK + with StrepII affinity tag fused *sqrF* gene, Km^r^ | (Marcia et al. 2010b) |
| pDSQNNS | pDSK6CrtKm with StrepII affinity tag fused *sqrF* gene, Km^r^ | (Marcia et al. 2010b) |
| pDSQNNSV331A | V331A mutant SqrF protein expressing pDSQNNS, Km^r^ | This work |
| pDSQNNSV331F | V331F mutant SqrF protein expressing pDSQNNS, Km^r^ | This work |
| pDSQNNSI333A | I333A mutant SqrF protein expressing pDSQNNS, Km^r^ | This work |
| pDSQNNSI331F | I333F mutant SqrF protein expressing pDSQNNS, Km^r^ | This work |
| pDSQNNSF366A | F366A mutant SqrF protein expressing pDSQNNS, Km^r^ | This work |
| pDSQNNSF366Y | F366Y mutant SqrF protein expressing pDSQNNS, Km^r^ | This work |
| pDSQNNSCTD | C-terminal α-helix deletion mutant SqrF protein expressing pDSQNNS, Km^r^ | This work |

**Table S3 Primers used in the study.**

| Name | Sequence |
| --- | --- |
| onV331AF | 5’CTTCAAGGTCGAGCTGGCGTGCATCGTCGACGCCAAC3’ |
| onV331AR | 5’GTTGGCGTCGACGATGCACGCCAGCTCGACCTTGAAG3’ |
| onV331AE | 5’GGCGTCGACGATGCACG3’ |
| onV331FF | 5’GACCTTCAAGGTCGAGCTGTTCTGCATCGTCGACGCCAACG3’ |
| onV331FR | 5’CGTTGGCGTCGACGATGCAGAACAGCTCGACCTTGAAGGTC3’ |
| onV331FE | 5’GACCTTCAAGGTCGAGCTGT3’ |
| onI333AF | 5’GAGACCTTCAAGGTCGAGCTGGTCTGCGCCGTCGACGCCAACGATACGGGCATGCTG3’ |
| onI333AR | 5’CAGCATGCCCGTATCGTTGGCGTCGACGGCGCAGACCAGCTCGACCTTGAAGGTCTC3’ |
| onI333AE | 5’GTTGGCGTCGACGGCGCAGA3’ |
| onI333FF | 5’GAGACCTTCAAGGTCGAGCTGGTCTGCTTCGTCGACGCCAACGATACGGG  CATGCTG3’ |
| onI333FR | 5’CAGCATGCCCGTATCGTTGGCGTCGACGAAGCAGACCAGCTCGACCTTGA  AGGTCTC3’ |
| onI333FE | 5’CAAGGTCGAGCTGGTCTGCT3’ |
| onF366AF | 5’CACTGGATGAAGCTCGGCGCCGAGTGGTGGTATCTGC3’ |
| onF366Ar | 5’GCAGATACCACCACTCGGCGCCGAGCTTCATCCAGTG3’ |
| onF366Ae | 5’CAGATACCACCACTCGGC3’ |
| onF366YF | 5’CACTGGATGAAGCTCGGCTACGAGTGGTGGTATCTGC3’ |
| onF366YR | 5’GCAGATACCACCACTCGTAGCCGAGCTTCATCCAGTG3’ |
| onF366YE | 5’GCAGATACCACCACTCGT3’ |
| onCTDF | 5’CAACTGGGTGCTGCCCTCGACATGATGATGACGGTTCTTCCACTGGATGAAG3’ |
| onCTDR | 5’CTTCATCCAGTGGAAGAACCGTCATCATCATGTCGAGGGCAGCACCCAGTTG3’ |
| onCTDE | 5’GAAGAACCGTCATCATC3’ |


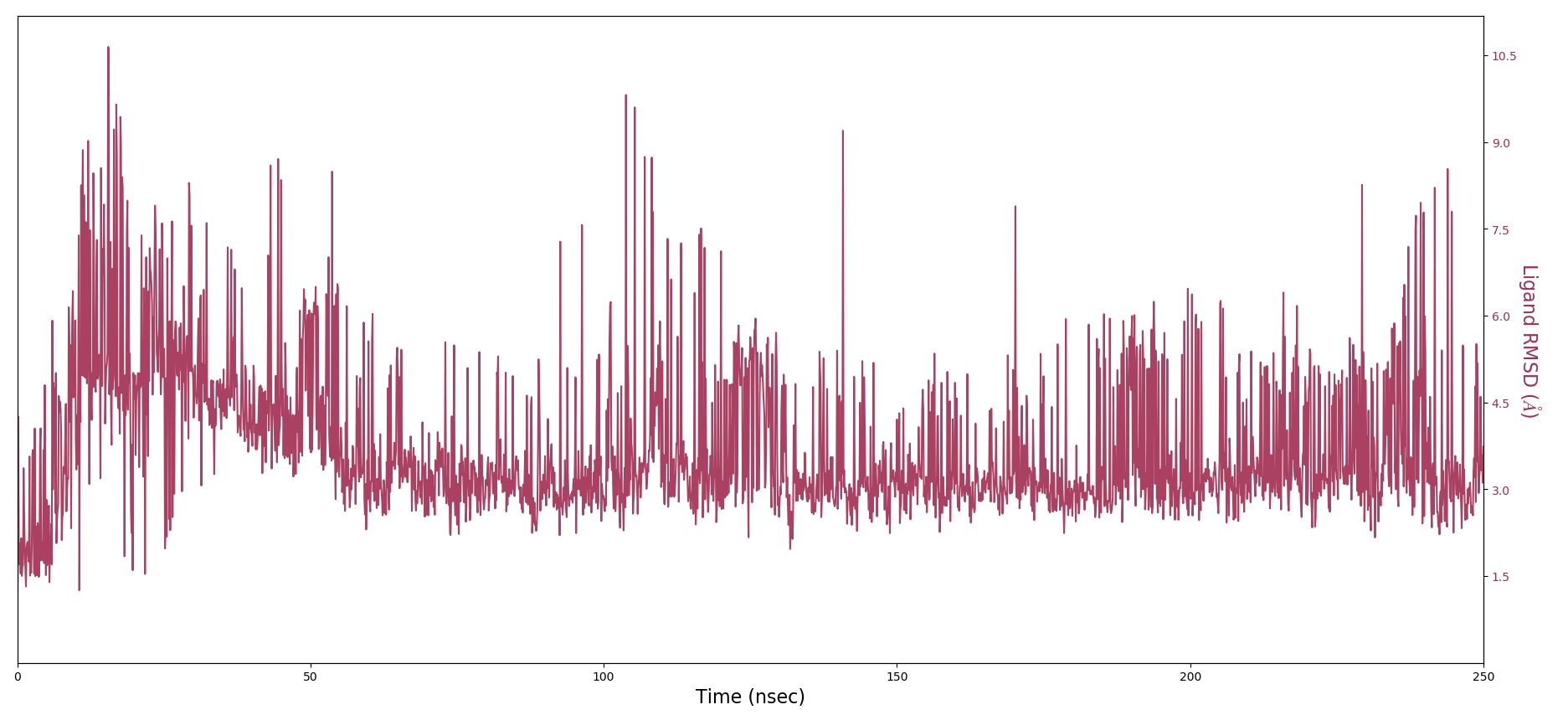


**Figure S1.** The root mean square deviation (RMSD) value of the ligand in relation to the starting position following fitting of the whole complex to the protein backbone.

**
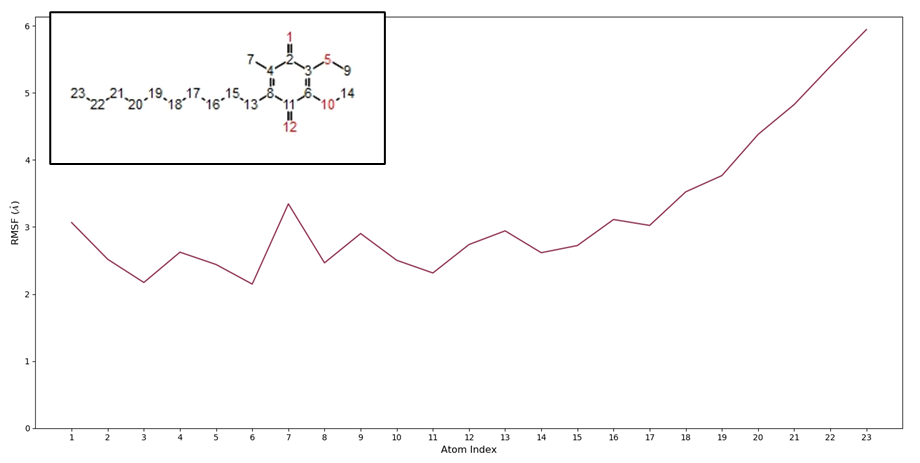
**

**Figure S2.** The root mean square fluctuation value of the heavy atoms in the ligand following the fitting of the complex to the protein backbone. The small figure illustrates the atom numbering.


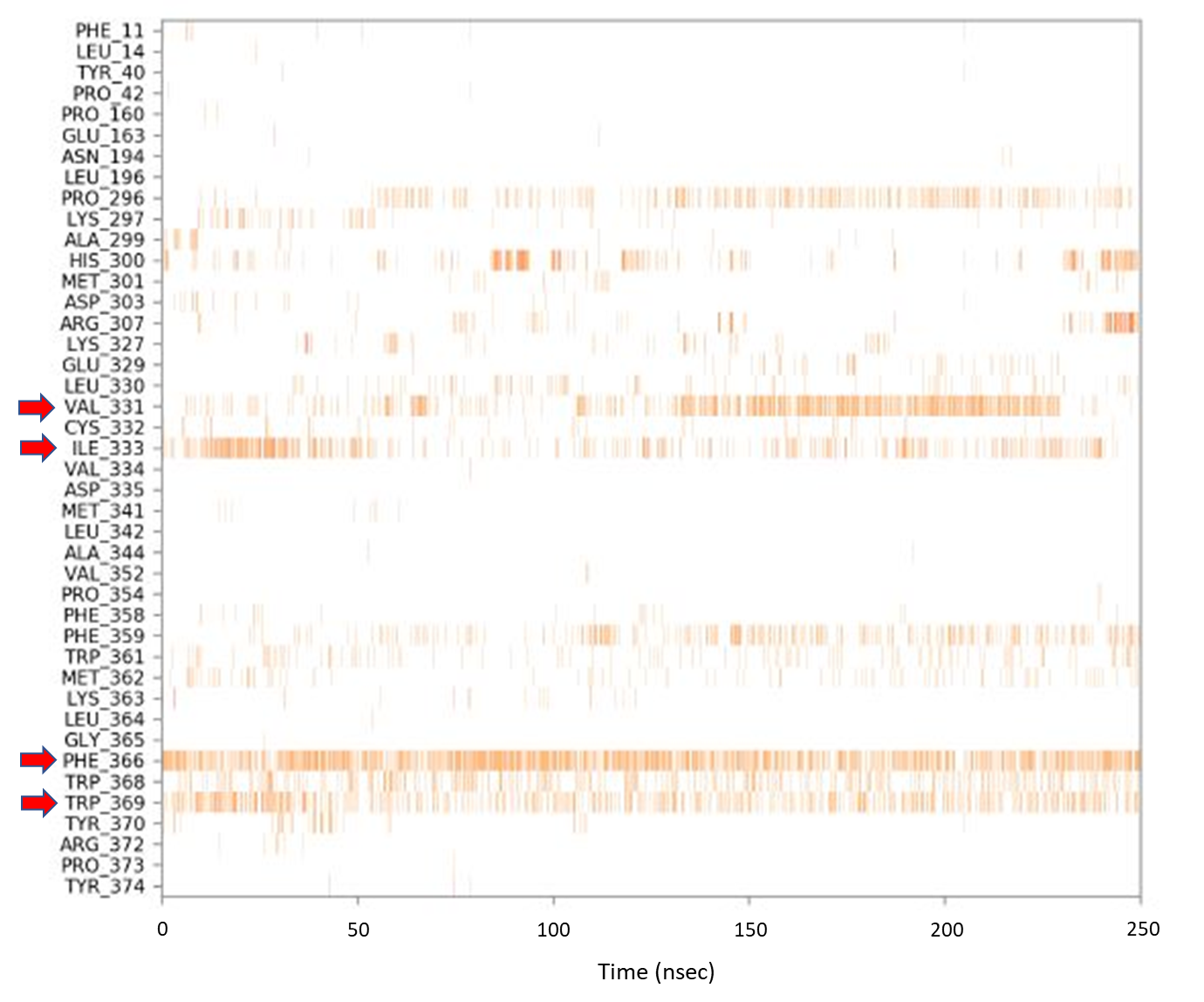


**Figure S3.** Timeline representation of the protein-ligand interactions in the simulated dUQ binding TrSqrF structures. The TrSqrF residues that interact with the ligand in each trajectory frame are depicted. The orange line denotes the specific contact between the amino acid and the ligand. The intensity of the shade of orange represents the number of specific contacts made by a residue with the dUQ. The red arrows indicate the quinone binding amino acids.


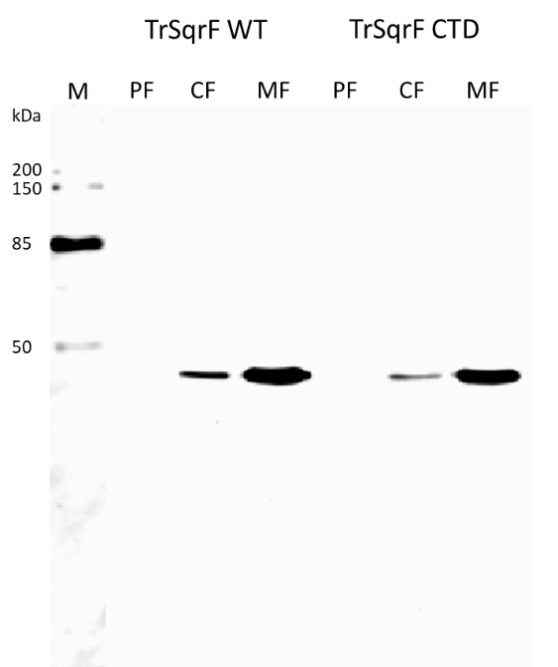


**Figure S4.** Cellular localization of the wild-type and the C-terminal α-helix deletion (CTD) TrSqrF mutants. Western blot analysis was used to detect TrSqrF proteins in the cell fraction samples using a Strep II tag-specific antibody (PF: periplasmic fraction; CF: cytoplasmic fraction, MF: membrane fraction). The first lane was loaded with a protein molecular weight marker (M).


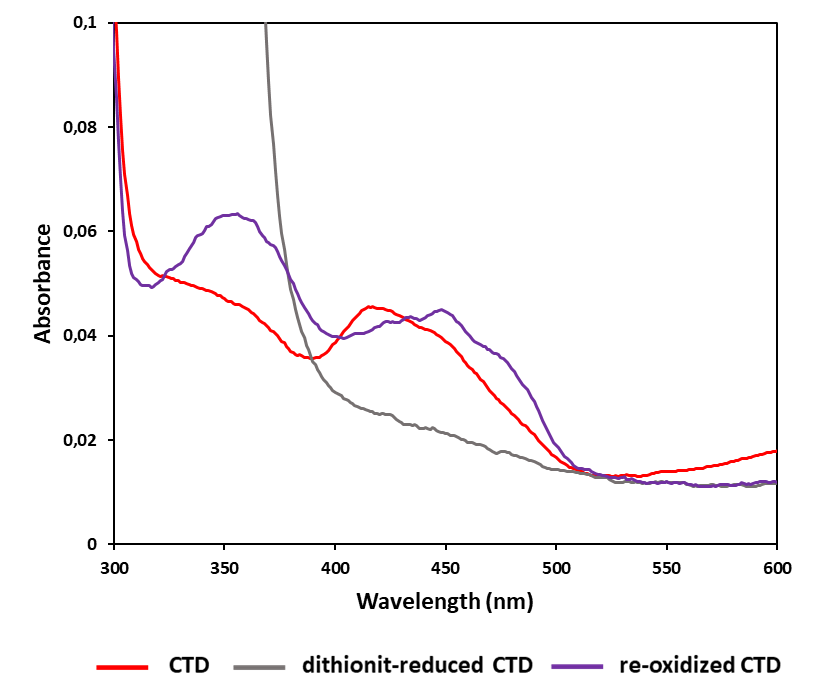


**Figure S5.** UV-visible absorption spectra of the purified, oxidized and reduced CTD mutant TrSqrF. The absorption spectrum of aerobically purified CTD mutant (10 µM) (red-colored) demonstrates the characteristically lower 360 and 448 nm peaks which are typical for reduced proteins. The absorption spectrum of the CTD mutant reduced with 1 mM dithionite (gray colored) revealed the disappearance of characteristic peaks of oxidized FAD molecules. Re-oxidation of the CTD mutant in the presence of 100 µM DQ (purple colored) exhibiting representative absorption peaks of oxidized state proteins.


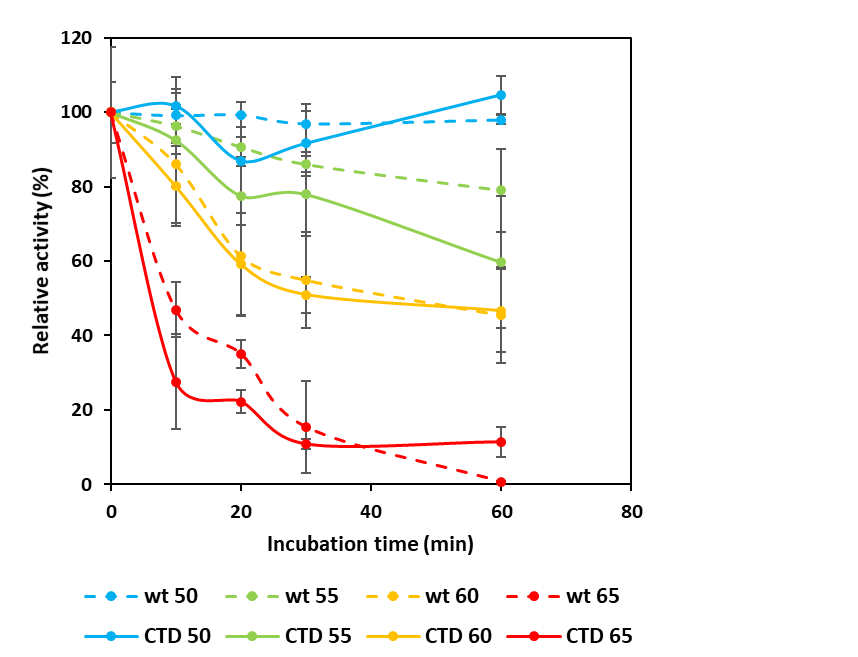


**Figure S6.** Effect of temperature on the stability of the wild-type enzyme (wt, dashed lines) (Duzs et al. 2018) and the C-terminal α-helix deletion variant of TrSqrF (CTD, solid lines) in this study. The enzyme samples were incubated at temperatures of 50 °C (blue lines), 55 °C (green lines), 60 °C (yellow lines), and 65 °C (red lines) for 60 minutes. Sulfide-dependent dUQ reducing activities of the treated samples were determined applying the standard activity assay at 25 °C.
